# Resistance to clinically important antibiotics and reduced susceptibility to disinfectants in the pandemic ST398 methicillin-susceptible *Staphylococcus aureus* from Austria

**DOI:** 10.3389/fmicb.2026.1734430

**Published:** 2026-04-21

**Authors:** Adrienn Gréta Tóth, Olga Makarova

**Affiliations:** 1 Department of Animal Breeding and Genetics, Institute for Animal Breeding, Nutrition and Laboratory Animal Science, University of Veterinary Medicine, Budapest, Hungary; 2 Centre for Bioinformatics, University of Veterinary Medicine, Budapest, Hungary; 3 Centre for Veterinary Public Health and One Health, VetMedUni Vienna, Vienna, Austria

**Keywords:** AMR, biocide resistance, community, MSSA, ST398

## Abstract

**Introduction:**

*Staphylococcus aureus* is a frequent coloniser and pathogen of humans and animals. The prevalence of human-associated methicillin-susceptible *Staphylococcus aureus* (MSSA) belonging to the pandemic sequence type (ST) 398 has been increasing globally; however, there are regional variations, with a high prevalence in France and a low prevalence in neighbouring Germany. Transmission within the community is thought to play an important role in its spread, as well as the presence of the *scn* gene, a marker of human adaptation.

**Methods:**

Environmental swabs were plated on Columbia sheep blood agar. A hemolytic isolate was identified as *S. aureus* by MALDI–ToF, and was tested for antimicrobial susceptibility to antibiotics using the Vitek 2 system and to disinfectants via broth microdilution. It was whole-genome sequenced using Illumina and bioinformatically analysed with respect to antimicrobial resistance genes, virulence-associated genes, ST, *spa* type, plasmids, prophages, and phylogenetic relationships with 273 other MSSA ST398 strains.

**Results:**

An MSSA strain belonging to ST398, of a yet-undescribed *spa* type (succession of repeat codes: 08-83-34-25), was isolated from a household in Vienna, Austria. The isolate was phenotypically classified as resistant to clindamycin and erythromycin, and as susceptible to levofloxacin under increased exposure, according to EUCAST guidelines. In addition, it had reduced susceptibility to the disinfectants benzalkonium chloride and chlorhexidine. Whole-genome sequencing revealed the presence of antimicrobial resistance and virulence-associated genes, including the *ermT* gene, located on a plasmid, and the *scn* gene, found on the StauST398 prophage. The isolate clustered most closely with human- and animal-derived isolates from France, almost all of which contained the phage-associated *scn* gene.

**Discussion:**

These preliminary results, based on a single isolate, demonstrate that an antibiotic-resistant *S. aureus* with potentially mobile antimicrobial resistance and virulence-associated genes belonging to MSSA ST398 is present in Austria. Further expanded surveillance efforts are needed to accurately assess its prevalence in the community. The reduced susceptibility to disinfectants in this strain is concerning, as it may negatively affect mitigation efforts. Its relatedness to other European isolates from humans and animals calls for a One Health approach to surveillance.

## 1 Introduction

*Staphylococcus aureus* is a commensal and opportunistic pathogen that colonises the skin and mucosal membranes of approximately 30% of the healthy human population and is also highly prevalent in animals (Haag et al., 2019; Matuszewska et al., 2020). The pandemic lineage of the sequence type (ST) 398 has received considerable attention due to its widespread distribution among livestock and humans (Wulf and Voss, 2008). Based on genomic properties and phage-carriage patterns, this lineage is further divided into livestock-associated (LA) methicillin-resistant *S. aureus* (MRSA) and human-associated methicillin-susceptible *S. aureus* (MSSA) ST398 (Price et al., 2012). MRSA ST398 has typically been linked to infections in humans who have had contact with livestock (Van Loo et al., 2007; Witte et al., 2007). In contrast, MSSA ST398 strains have been commonly identified in humans lacking direct animal contact (Uhlemann et al., 2012; Valentin-Domelier et al., 2011). The presence of an immune evasion cluster (IEC) on the φSa3 prophage, which encodes the staphylococcal complement inhibitor (SCIN) gene *scn*, which mediates specific affinity to human skin keratinocytes, is believed to underlie the success of this lineage in humans (Thammavongsa et al., 2015; Uhlemann et al., 2012), with the *scn* gene being considered a marker of human host adaptation (Price et al., 2012). In humans, MSSA ST398 isolates have often been associated with severe bloodstream, bone, and joint infections (Bouiller et al., 2020; Valour et al., 2014) and are frequently resistant to erythromycin and clindamycin (Uhlemann A. C. et al., 2013) due to the presence of the *ermT* gene (Chroboczek et al., 2013). The prevalence of MSSA ST398 varies across regions and isolation sources, with France and China reporting, particularly high rates of 5.5–26.6% among clinical isolates (Bouiller et al., 2020), while a study of MSSA ST398 of the *spa* type t571 in Germany, from both infections and nasal colonisation in healthy humans in the community, showed a low prevalence of 0.13–0.14% (Cuny et al., 2013). Although MSSA isolates are not routinely genotyped as part of national surveillance programmes, making it difficult to assess the prevalence of ST398, individual studies suggest that globally MSSA ST398 has been on the rise and has been specifically described in numerous European countries, such as Belgium, Denmark, Greece, Ireland, Italy, Portugal, and Spain (Bouiller et al., 2020; Salgueiro et al., 2023; Soavi et al., 2010; Valentin-Domelier et al., 2011). Transmission in the community is thought to play a particularly important role in the spread of MSSA ST398 (Annavajhala et al., 2022; David et al., 2013; Uhlemann A. et al., 2013), and the environmental contamination of households has previously been identified as a primary reservoir of *S. aureus* in the community (Davis et al., 2012; Knox et al., 2012; Uhlemann et al., 2011). Biocides, such as chlorhexidine (Sharaf et al., 2025) and benzalkonium chloride (Bondurant et al., 2020), are frequently used for decontamination, yet few studies have investigated the tolerance of *S. aureus* to these biocides (Kernberger-Fischer et al., 2018).

In this study, we report an MSSA ST398 strain of a previously undescribed *spa* type, isolated from a household in Austria that combines phenotypic resistance to clinically important antibiotics with reduced susceptibility to common biocides. Additionally, we provide an extensive genomic analysis of virulence factors, antimicrobial resistance determinants, mobile genetic elements (MGEs), and phylogenetic relationships with MSSA ST398 isolates from different countries and both human and animal hosts, with a particular focus on the presence of the *scn* gene, which is considered a marker of human adaptation.

## 2 Materials and methods

### 2.1 Bacterial culture and antimicrobial susceptibility testing

Environmental swabs (*n* = 7) from high-frequency touch surfaces (door handles, faucets, and sinks) in a household without pets in Vienna, Austria, were collected in March 2023 as part of a pilot citizen science project, suspended in 1 mL of 0.99% sterile saline, and stored at 4 °C for 1 week until further processing. In total, 100 μL of the sample was spread on Columbia Blood Agar with Sheep Blood Medium (Thermo Scientific™ Waltham, MA) using sterile glass beads and incubated aerobically for 24 h at 37 °C. Colonies of different morphologies (one per type) were identified to a species level by MALDI–ToF MS (MALDI Biotyper® (MBT), Billerica, MA) following the manufacturer’s instructions. All bacterial cultures were grown in Mueller–Hinton (MH) broth (Oxoid, Basingstoke, UK) at 37 °C in an orbital shaker at 200 rpm for 18 h, unless specified otherwise. Cryostocks of *Staphylococcus aureus* strain 257277 were prepared by adding sterile glycerol to the overnight culture to a final concentration of 20% and stored at −80 °C.

For antibiotic susceptibility testing (AST), the VITEK® 2 system (AES software, bioMérieux, Marcy l’Étoile, France) and AST-P654 (bioMérieux) cards were applied, according to the manufacturer’s instructions. EUCAST clinical breakpoints were used (EUCAST, 2023).

For biocide susceptibility testing, minimum inhibitory concentrations (MICs) were determined via broth microdilution (Wiegand et al., 2008). Briefly, 2-fold dilutions of benzalkonium chloride (BAC) (Sigma-Aldrich, St. Louis, MO, United States) or chlorhexidine (CHX) digluconate (Sigma-Aldrich, St. Louis, MO, United States) in the final concentration range of 0.25–16 μg/mL were dispensed into polystyrene 96-well plates with conical bottoms (Sarstedt GmbH, Nümbrecht, Germany) containing 100 μL (final volume) of MH broth. To prepare the inoculum, overnight cultures were adjusted with MH broth to an optical density (OD600) of 0.5 and further diluted 1:100. Five microliters of the final inoculum was added to each well. The plates were incubated in a humidity chamber at 37 °C for 16–20 h and inspected visually for growth. The disinfectant susceptibility testing reference strain *Staphylococcus aureus* ATCC 6538 (Makarova et al., 2017) was used as a control. Each strain was tested in triplicate.

### 2.2 Whole-genome sequencing and bioinformatic analyses

Whole-genome sequencing (WGS) was performed by MicrobesNG (Birmingham, UK). Cells were harvested by centrifugation of the overnight culture and resuspended in DNA/RNA Shield (Irvine, CA). They were then lysed using TE buffer containing lysozyme (Solon, OH), Metapolyzyme (St. Louis MO), and RNase A (ITW Reagents, Spain), followed by treatment with proteinase K (VWR Chemicals, Ohio, United States) and SDS (Sigma-Aldrich, Missouri, United States) according to the MicrobesNG strain submission procedures. DNA was extracted using SPRI (solid-phase reversible immobilisation) beads, resuspended in EB buffer (10 mM Tris–HCl, pH 8.0), quantified with the Quant-iT dsDNA HS (Thermo Fisher Scientific, MA, United States) assay in an Eppendorf AF2200 plate reader (Eppendorf UK Ltd., UK), and diluted as appropriate by MicrobesNG. Genomic DNA libraries were prepared with the Nextera XT Library Prep Kit (Illumina, San Diego, CA, United States) according to the manufacturer’s instructions, with two modifications: the input DNA amount was doubled, and the PCR elongation time was extended to 45 s. Libraries were sequenced on an Illumina NovaSeq 6000 (Illumina, San Diego, CA, United States) with a 250 bp paired-end protocol at a coverage of 30×. The raw short reads are uploaded to the National Center for Biotechnology Information (NCBI) Sequence Read Archive (SRA) under the accession number PRJNA1136839.

Quality-based filtering and trimming of the pre-trimmed short reads were performed using TrimGalore (v0.6.7^1^), setting 20 as a quality threshold and a minimal length of 50 bp. The remaining reads were assembled into contigs using MEGAHIT (v1.2.9) (Li et al., 2015) with default settings. Genome assembly quality was assessed using QUAST (v5.3.0) (Gurevich et al., 2013), and genome annotation was performed with Prokka (v1.14.6) (Seemann, 2014). To assess the sequencing depth, trimmed reads were aligned to the *Staphylococcus aureus* reference genome (GCF_000013425.1) obtained from NCBI RefSeq (O’Leary et al., 2015) using Bowtie2 (v2.5.3) (Langmead and Salzberg, 2012) and the functions of SAMtools (v1.19) (Li et al., 2009). MLST was performed with the mlst software^2^ using the PubMLST database (Jolley et al., 2018). *Spa* typing was executed with SpaTyper (v0.3.3)^3^ using the sequences found on the Ridom SpaServer.^4^ Virulence genes were screened using Abricate (v1.0.1)^5^ with the Virulence Factor Database (VFDB) downloaded on 04/06/2024 (Chen et al., 2016). All possible open reading frames (ORFs) were predicted from the contigs using Prodigal (v2.6.3) (Hyatt et al., 2010). The protein-translated ORFs were aligned to the ARG (antimicrobial resistance genes) sequences of the Comprehensive Antibiotic Resistance Database (CARD, v3.2.9) (Jia et al., 2017; McArthur et al., 2013) using the Resistance Gene Identifier (RGI, v6.0.3) with Diamond (Buchfink et al., 2014). The ORFs classified as “Perfect” or “Strict” were further filtered for 90% identity and 90% coverage. The ARG content was further elucidated using ResFinder (v4.5.0) (Zankari et al., 2012) with the ResFinder (v2.3.2) and the DisinFinder database (v2.0.1). BacMetSearch^6^ was run on the BacMet database (v2.0) (Pal et al., 2014) with Diamond (Buchfink et al., 2014) to assess further biocide resistance genes. The AMRrules tool^7^ was used for the interpretation of AMR genotype data. The plasmid origin probability of the contigs was estimated using PlasFlow (v.1.1) (Krawczyk et al., 2018). The prophage content of the assembled contigs was predicted using VirSorter2 (v2.2.4) (Guo et al., 2021). The findings were filtered for dsDNA phages and ssDNAs. Phage-associated host contamination was removed using CheckV (v1.0.1, with database v1.5) (Nayfach et al., 2020). The resulting contigs were aligned to the nt_viruses database (v5) using BLAST (Basic Local Alignment Search Tool) (Altschul et al., 1990). The annotation of the prophages was performed with Prokka (v1.14.6) (Seemann, 2014). All data management procedures, analyses, and plotting were performed in the R environment (R Core Team, 2023).

### 2.3 Phylogenetic analysis

A metadata table of 4,029 BioSamples was downloaded from the NCBI Sequence Read Archive (SRA) (Leinonen et al., 2011) after searching for the following keywords: “*Staphylococcus aureus* ST398,” “*Staphylococcus aureus* CC398” (accessed on 05/06/2024). BioSamples from three BioProjects (PRJNA763220, PRJNA347471, and PRJNA901657) were immediately selected to be included due to containing sequences from MSSA isolates. The remaining samples were filtered as follows. To ensure consistent phylogenetic analyses, BioSamples with sequencing depth similar to that of our sample (base numbers of 100,000,000 to 300,000,000) were selected, which may have resulted in the exclusion of genomes of high coverage (no record of the number of excluded genomes was made). From the remaining BioProjects, the isolation hosts were screened, and pigs were excluded due to their high MRSA carriage rates. All data management procedures were performed with R (v4.1.2) (R Core Team, 2023). From the remaining BioProjects, those that were not explicitly indicated to contain MRSA strains were selected based on their project descriptions. BioSamples from this subset of BioProjects and the three pre-selected BioProjects were further filtered by re-sorting for ST398 and checking for the presence of *mecA*, *mecB*, *mecC,* or *mecD* using the same bioinformatic analysis steps described above. Thus, a final set of 273 BioSamples that exclusively contained MSSA ST398 was obtained and used for further analysis. Three outgroups (*Bacillus subtilis*—GCF_000009045.1, *Macrococcus epidermidis*—GCF_024205965.1, *Staphylococcus argenteus*—GCF_000236925.1) were added to the sequence set from NCBI RefSeq (Haft et al., 2018).

Core genes were selected if they appeared in at least 99% of all genomes. For the core-genome analysis, MAFFT (v7.490) (Katoh et al., 2002) was used for multiple sequence alignment. Single-nucleotide polymorphisms (SNPs) were identified using snp-sites (v2.5.1) (Page et al., 2016). The best substitution model was selected using functions from the phangorn (v2.11.1) package (Schliep, 2011) based on the Bayesian information criterion (BIC) to calculate pairwise evolutionary distances between sequences. Pangenome analysis was based on the binary gene presence–absence matrix generated by Panaroo (v1.5.0) (Tonkin-Hill et al., 2020) in strict mode with default settings, with pairwise distances between genomes calculated using the binary distance metric. Initial neighbour-joining (NJ) trees were inferred for the core-genome and pangenome based on the pairwise distance matrices using ape (v5.8.1) (Paradis and Schliep, 2019) and phangorn (v2.11.1) (Schliep, 2011) packages in R (v4.1.2) (R Core Team, 2025). Maximum-likelihood (ML) trees were created by optimising the initial NJ trees [branch length, topology, and among-site rate heterogeneity (gamma distribution)] under the GTR model for the core-genome and the ER model for the pangenome using phangorn (Schliep, 2011). Bootstrap values were calculated using 100 iterations. Visualization was performed using the ggtree and ggtreeExtra packages (Xu et al., 2021; Yu, 2020). Based on both trees, 20 BioSamples that clustered the closest to our sample were bioinformatically analysed to assess the presence of gene *scn* [potential marker for human adaptation (Howden et al., 2023)] on their phages using the methods described above. Average nucleotide identity (ANI) was calculated using FastANI (v1.34) (Jain et al., 2018). The query genome was compared against all genomes that clustered with the query genome in the phylogenetic analyses, and ANI values were computed using default FastANI parameters. Pairwise SNP distances were calculated from the core SNP alignment for the same subset of closely related genomes as above. SNP distances were computed as Hamming distances and visualised as a heatmap using ggplot2 (Wickham, 2011).

## 3 Results

### 3.1 Isolate characterisation

**Figure 1:**
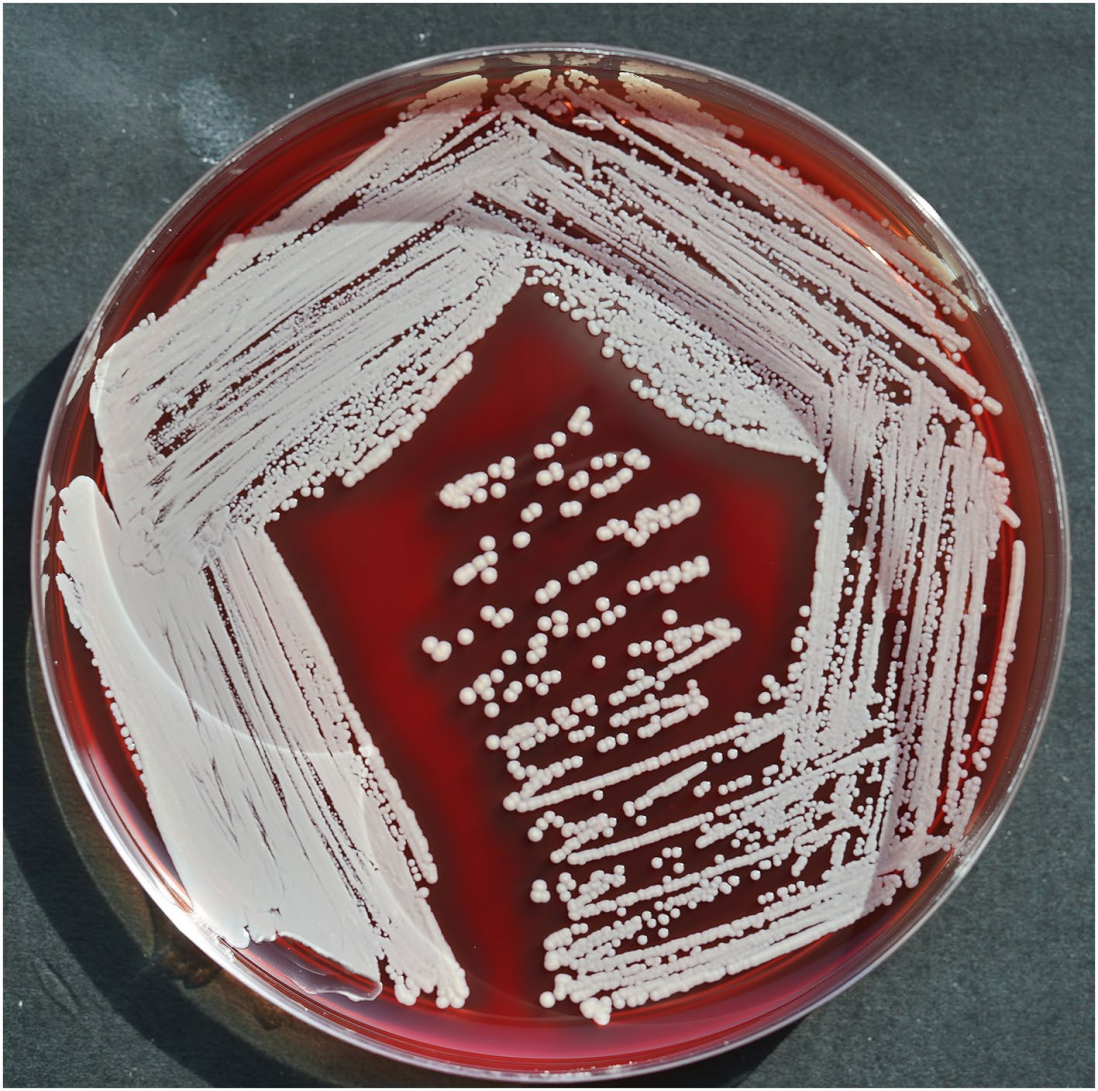
*S. aureus* MSSA ST398 strain 257277 exhibiting a beta-hemolytic ability on Columbia Blood Agar plates with Sheep Blood Medium.

*Staphylococcus aureus* strain 257277 was isolated from a household in Vienna, Austria, as part of a pilot citizen science project, via nonselective culturing on Columbia blood agar, on which it formed white hemolytic colonies (Figure 1). Lower levels of staphyloxanthin production are typical for livestock-associated MRSA ST398 (Pomba et al., 2010; Busche et al., 2018). Nonetheless, MLST, WGS, and AST analyses revealed that the isolate is methicillin-susceptible and belongs to the pandemic sequence type 398 (MSSA ST398). The genome assembly of the WGS data consisted of 36 contigs of at least 1 kb in size (421 contigs in total) with a total assembly length of 2,802,664 bp and an N50 value of 140,335 bp. Genome annotation predicted 2,468 coding sequences (CDSs). Further genotyping showed that the isolate is of a previously undescribed *spa* type (succession of repeat codes: 08-83-34-25). The *spa* sequence (Supplementary Table S1) was detected on a contig of 63,545-bp length unambiguously, without any truncation, frameshift, or low-quality warning (flag = 0), with a coverage of 79.9452.

In summary, MSSA of pandemic lineage ST398 and an undescribed *spa* type was identified in a community setting in Austria for the first time.

### 3.2 Antimicrobial resistance

**Figure 2:**
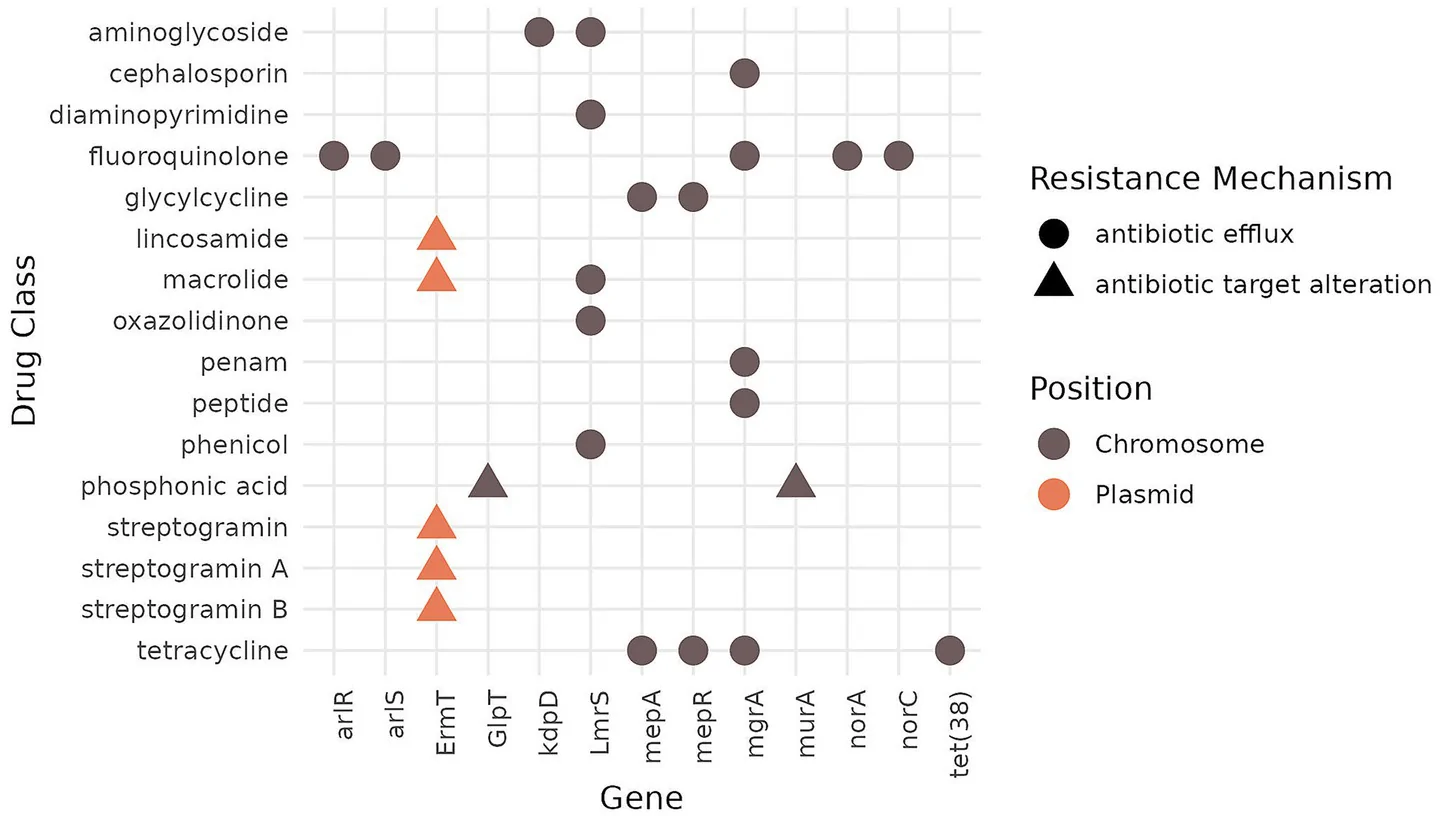
Antibiotic resistance genes (ARGs), potentially affected drug classes, resistance mechanisms, and ARG position in the genome based on the analysis of the WGS of the MSSA ST398 strain 257277 using the CARD database. Please note that, except for *ermT*, all ARGs listed here correspond to wild-type core genes in *S. aureus* present in susceptible phenotypes according to the expert-curated database AMRules (https://github.com/AMRverse/AMRrules/blob/main/rules/Staphylococcus_aureus.tsv, accessed on 06/03/2026). Mutations *glpT* A10*0V/F3I* and *murA* D27*8E/E291D* were also associated with phenotypical fosfomycin susceptibility.

WGS analysis of antimicrobial resistance genes (ARGs) showed the presence of 14 ARGs using the CARD database (Jia et al., 2017; McArthur et al., 2013), whereas only one, *ermT,* was identified based on the ResFinder database (Zankari et al., 2012) using the same thresholds (data not shown). The majority of the resistance genes predicted using the CARD database were associated with antibiotic efflux and were chromosomally encoded: *arlR* and *arlS* (fluoroquinolone resistance), *kdpD* (aminoglycoside resistance), *lmrS* (aminoglycoside, diaminopyrimidine, macrolide, oxazolidinone, and phenicol resistance), *mepA* and *mepR* (glycylcycline and tetracycline resistance), *mgrA* (resistance to cephalosporin, fluoroquinolone, penam, peptide, and tetracycline groups of antibiotics), *norA* and *norC* (fluoroquinolone resistance), and *tet*(38) (tetracycline resistance). Nonetheless, a further analysis using the expert-curated interpretive standards for AMR genotypes “AMR rules”^8^ for *S. aureus* revealed that all of these genes [*arlR*, *arlS, kdpD, lmrS*, *mepA*, *mepR*, *mgrA*, *norA*, *norC,* and *tet*(38)] correspond to wild-type susceptible phenotypes and do not contribute to phenotypic clinical resistance. Other ARGs identified using the CARD database were associated with the resistance mechanism of antibiotic target alteration: *glpT* and *murA* (phosphonic acid resistance) and *ermT* (resistance to lincosamide, macrolide, and streptogramin groups of antibiotics). However, the specific gene variants of *glpT* (with A100V and F3I mutations) and *murA* (with D278E and E291D mutations) present in this strain are associated with phenotypic susceptibility (Salgueiro et al., 2023; Fu et al., 2016). Only one ARG, *ermT*, was predicted to originate from a plasmid. No ARGs were associated with phages (Figure 2). Antimicrobial susceptibility testing using the Vitek 2 system with a panel of clinically relevant antibiotics confirmed phenotypic resistance only to clindamycin and erythromycin, while susceptibility to fluoroquinolones (levofloxacin) was determined as “susceptible to increased exposure” (“I,” formerly classified as intermediate), according to EUCAST interpretative criteria. The isolate appeared to be susceptible to aminoglycosides, diaminopyrimidines, glycylcyclines, macrolides, oxazolidinones, penams, peptide antibiotics, phosphonic acid, tetracyclines, cefoxitin, fusidic acid, mupirocin, and rifampicin (Table 1).

**Table 1:** Antimicrobial susceptibility testing to antibiotics by the VITEK2 system using the P654 panel and interpreted according to EUCAST 2023 clinical breakpoints.

Antibiotic class	Antibiotic	MIC, μg/mL	Interpretation
Cephamycins (beta-lactams)	Cefoxitin-screen	NEG	–
Penicillins (beta-lactams)	Benzylpenicillin	0.12	S
	Oxacillin	≤0.25	S
Aminoglycosides	Gentamicin	≤0.5	S
Fluoroquinolones	Levofloxacin	0.5	I
**Macrolides**	**Erythromycin**	**≥8**	**R**
**Lincosamides**	**Clindamycin**	**0.25**	**R**
Oxazolidinones	Linezolid	2	S
Lipopeptides	Daptomycin	0.5	S
Glycopeptides	Teicoplanin	≤0.5	S
	Vancomycin	1	S
Tetracyclines	Tetracycline	≤1	S
Glycylcyclines	Tigecycline	≤0.12	S
Phosphonic acid	Fosfomycin	≤8	S
Fusidane (steroidal)	Fusidic acid	≤0.5	S
Carboxylic acid	Mupirocin	≤1	S
Rifamycins	Rifampicin	≤0.03	S
Diaminopyrimidines/sulphonamides	Trimethoprim/sulfamethoxazol	≤10	S

NEG, negative; R, resistant; I, susceptible to increased exposure; S, susceptible. Resistance to antibiotics is highlighted in bold.

**Figure 3:**
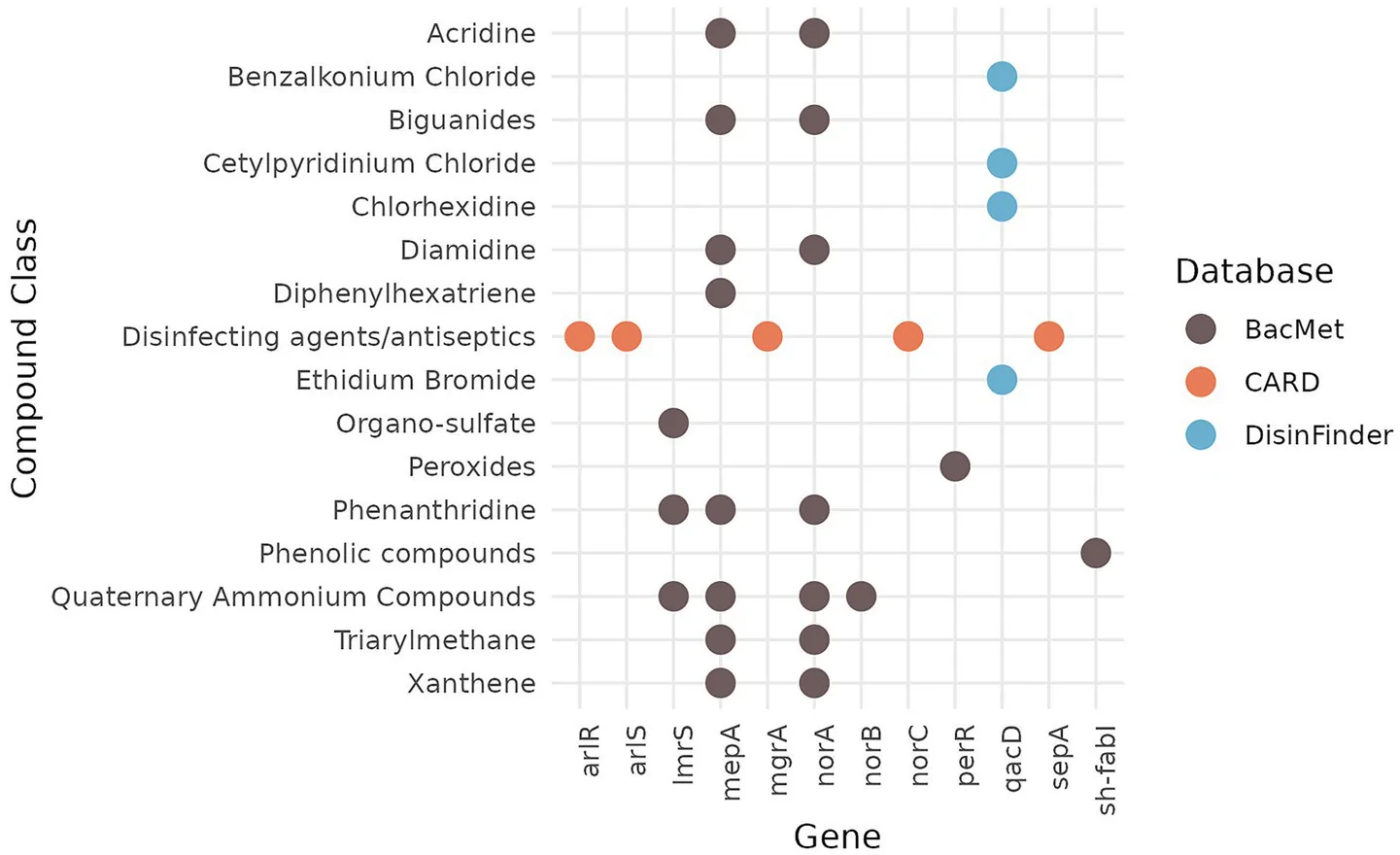
Resistance genes against disinfectants and biocides and disinfectant/biocide compound class identified in the WGS of MSSA ST398 strain 257277 using BacMet, DisinFinder and CARD databases. Colours represent the database screened to identify the given gene. Hits were filtered for a minimum identity rate of 90%. Please note that *arlR*, *arlS*, *lmrS*, *mepA*, *mgrA, norA*, *norB*, *norC* listed here correspond to wild-type core genes in *S. aureus* present in susceptible phenotypes according to the expert-curated database AMRules (https://github.com/AMRverse/AMRrules/blob/main/rules/Staphylococcus_aureus.tsv, accessed on 06/03/2026).

In addition, the genome was screened for ARGs potentially affecting susceptibility to disinfectants or biocides using the BacMet (Pal et al., 2014), CARD (Jia et al., 2017; McArthur et al., 2013), and DisinFinder (Zankari et al., 2012) databases, with 13 ARGs identified (Figure 3), none of which appeared to be mobile. The majority of the ARGs (*n* = 6) were identified using the BacMet database, while five were detected with the CARD database, and only one (*qacD*) was detected using the DisinFinder database. Interestingly, none of the identified ARGs overlapped between the three databases. While many of these genes (*arlR, arlS*, *lmrS*, *mepA*, *norA,* and *norC*) appear in both biocide/disinfectant and antibiotic resistance gene databases, they represent wild-type core genes in *S. aureus* according to the AMRrules^9^ (Figures 2, 3).

ARGs unique to biocide/disinfectant ARGs were *perR* (peroxides), *qacD* (benzalkonium chloride, cetylpyridinium chloride, chlorhexidine, and ethidium bromide), *sepA* (disinfectants/antiseptics), and *sh-fabI* (phenolic compounds). Since the isolate had a predicted *qacD* ARG associated with reduced susceptibility to benzalkonium chloride and chlorhexidine, which are commonly used surfactant/biocide and an antiseptic, respectively, we tested the susceptibility of *Staphylococcus aureus* strain 257277 to these antimicrobial agents and compared the MIC values to those of the disinfectant testing reference strain *Staphylococcus aureus* ATCC 6538. Indeed, the *S. aureus* strain 257277 had a lower susceptibility to BAC and CHX (2 μg/mL for both) than the biocide testing reference strain ATCC 538 (1 μg/mL for both) (Table 2).

**Table 2:** Antimicrobial susceptibility testing to biocides by broth microdilution.

Isolate	MIC, μg/mL	
	BAC	CHX digluconate
*S. aureus* MSSA ST398 strain 257277	**2**	**2**
*S. aureus* ATCC6538	1	1

BAC, benzalkonium chloride; CHX, chlorhexidine. Reduced susceptibility highlighted in bold.

In short, the MSSA isolate of the pandemic ST398 lineage encoded ARGs associated with resistance to antibiotics and biocides/disinfectants, was phenotypically resistant to macrolides and lincosamides (with the corresponding ARG, *ermT*, predicted to be located on a plasmid), and showed a reduced susceptibility to the QAC biocide BAC and the biguanide disinfectant CHX.

### 3.3 Virulence traits

**Figure 4:**
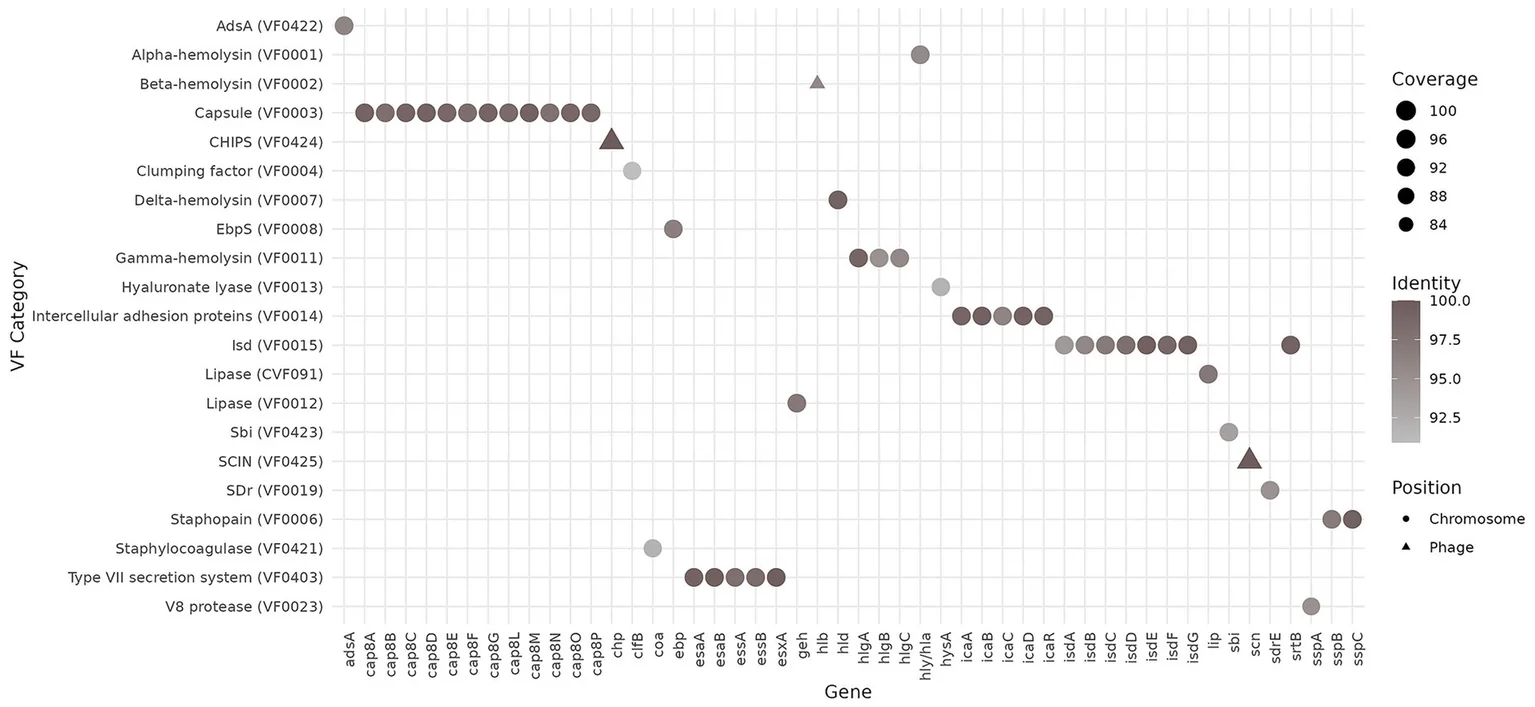
Virulence-associated genes (VAGs), virulence factor category, and VAG position in the genome of the MSSA ST398 strain 257277 were identified using the VFDB database. Identity and coverage rates are compared to the sequences of VFDB. VF, virulence factor.

A total of 50 virulence-associated genes (VAGs) were identified based on the VFDB (Chen et al., 2016) (Figure 4). Almost all of them reached at least 90% identity and 90% coverage compared to the sequences stored in the VFDB. The largest number of VAGs were associated with the capsule, adhesion, iron acquisition, and the type VII secretion system, as well as several alpha, delta, and gamma hemolysins, all encoded chromosomally. However, while no virulence genes were associated with plasmids, the *chp* gene (encoding the chemotaxis inhibitory protein CHIPS) and the *scn* gene (encoding staphylococcal complement inhibitor SCIN), both of which are involved in immune evasion, were found on a prophage. Based on the BLAST analysis of the phage, the StauST398-4 prophage was the most probable hit. Other genes that were present in the phage genome included: *xerC*, *dinG,* and *ssbA* (involved in DNA repair and recombination); *lexA* (SOS response regulator); *dut* (induction of *S. aureus* pathogenicity island), *clpP* (stress, virulence, and DNA repair regulator); and two beta-hemolysin genes (*hlb_1* and *hlb_2*). Indeed, the isolate demonstrated a beta-hemolytic activity on blood agar (Figure 1).

To sum up, the MSSA ST398 isolate encoded multiple virulence-associated genes, with beta-hemolysin and immune evasion-related genes located on a mobile genetic element (prophage).

### 3.4 Phylogenetic analysis

**Figure 5:**
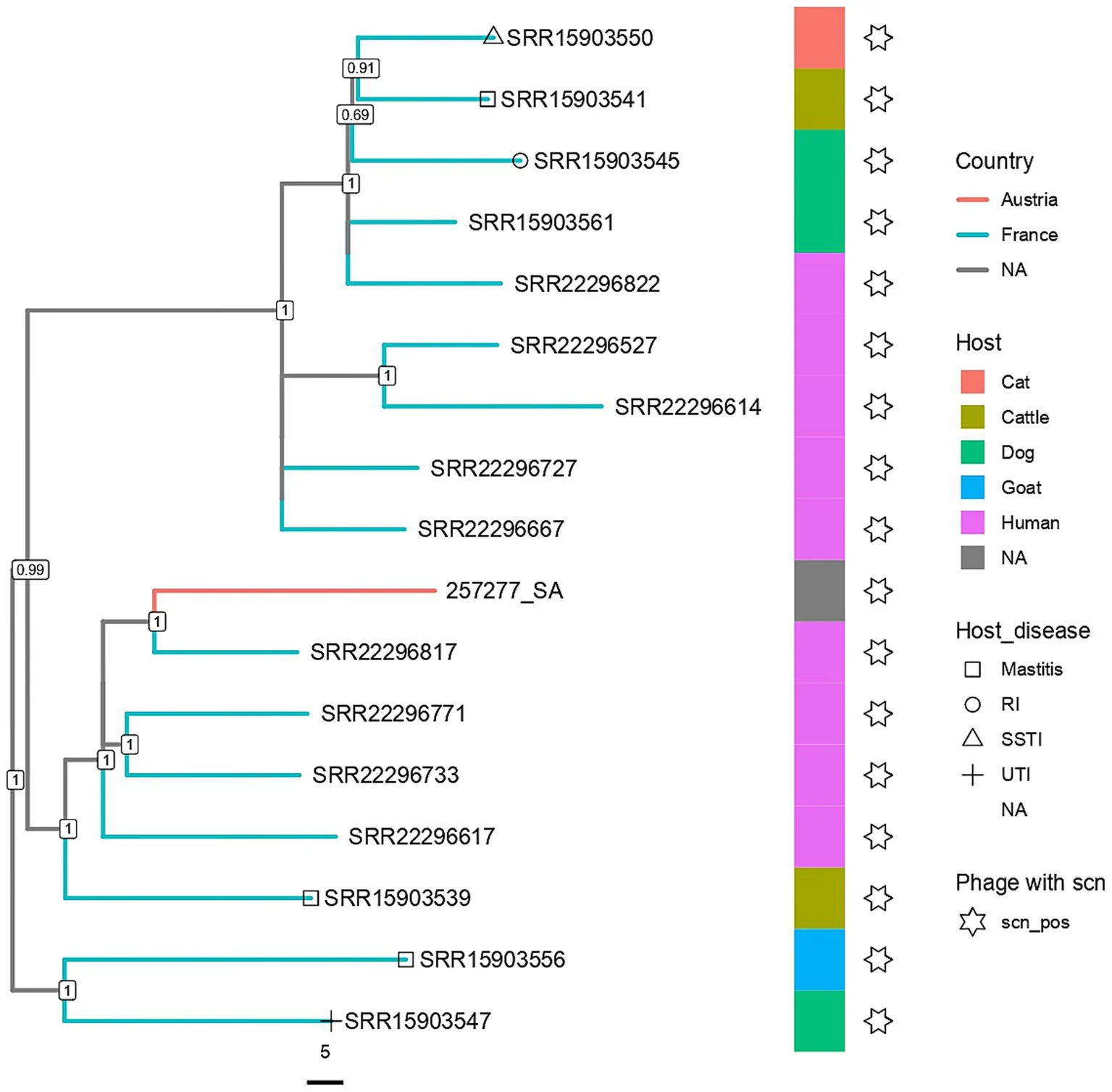
A zoom-in of the core-genome maximum likelihood tree. For the full tree, see Supplementary Figure S1. RI, respiratory infection; SSTI, skin and soft tissue infection; UTI, urinary tract infection.

**Figure 6:**
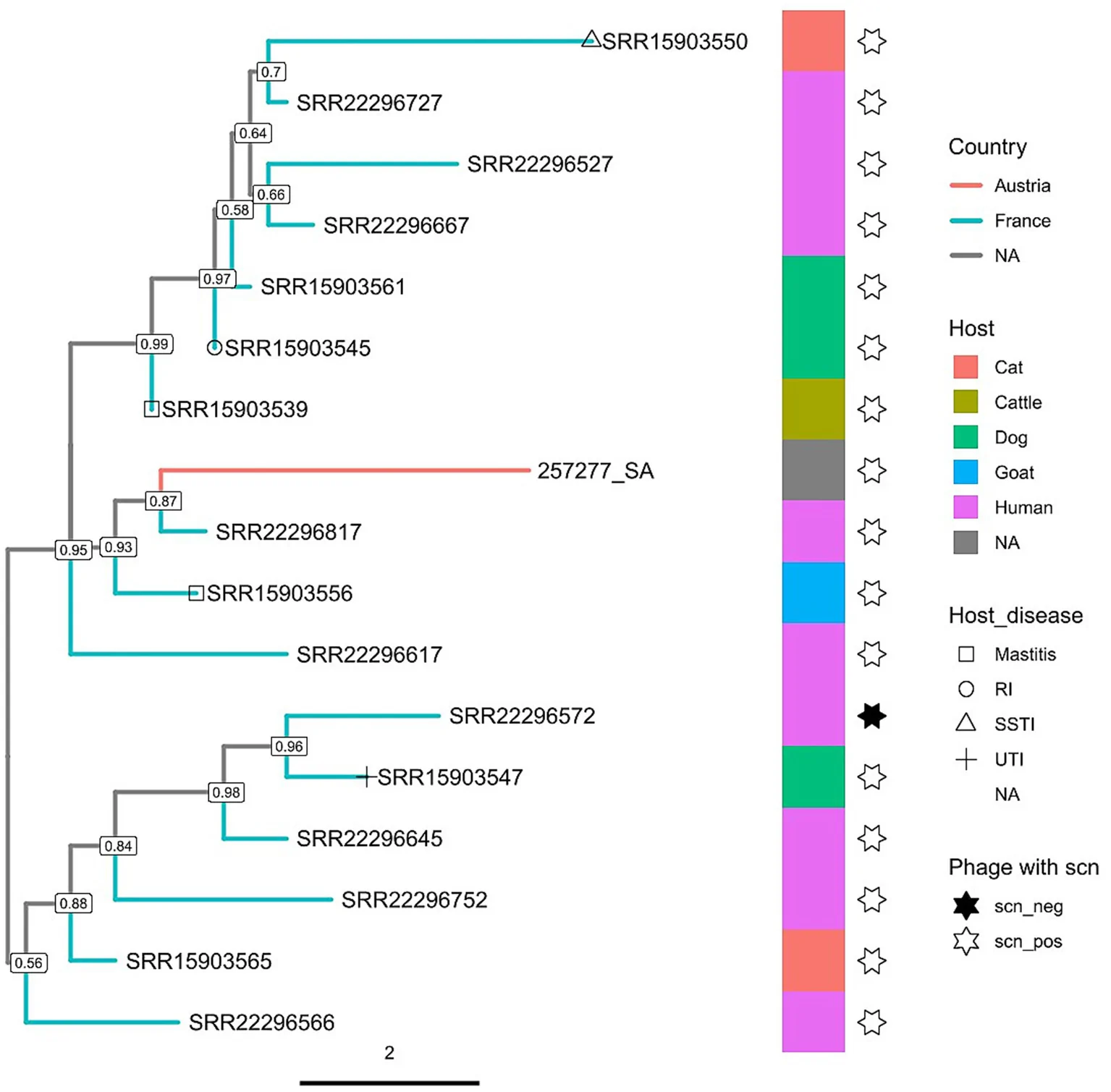
A zoom-in of the pangenome maximum likelihood tree. For the full tree, see Supplementary Figure S2. RI, respiratory infection; SSTI, skin and soft tissue infection; UTI, urinary tract infection.

Given that there are only a few reports of MSSA ST398 in Austria (Luxner et al., 2014) and considering its pandemic global nature, we next aimed to determine the relatedness of this isolate to other global MSSA ST398 strains. To achieve this, we constructed core-genome neighbour-joining (cgNJ) and pangenome neighbour-joining (pNJ) phylogenetic trees (Supplementary Figures S1–S4), which were further optimised for branch length, topology, and among-site rate heterogeneity using the maximum-likelihood method, resulting in core-genome Maximum-Likelihood (cgML) and pangenome Maximum-Likelihood (pML) trees (Figures 5, 6; Supplementary Figures S5, S6) with 273 MSSA ST398 strains derived from 15 BioProjects from 9 countries of 4 continents. Of these, 103 samples were derived from the United States, 90 from France, 67 from China, 3 from Martinique, 2 from Canada, 1 from Australia, 1 from the Dominican Republic, and 1 from Italy. The origins of the four samples were not included in the metadata. The strains were isolated from eight host species, including both humans and animals. Furthermore, six host disease types were registered. Within the studied genomes, a total of 3,653 genes were identified. Of these, 2,068 were core genes.

The cgML, pML, cgNJ, and pNJ trees were congruent and showed separate clades comprising isolates primarily from the United States, China, and France (Supplementary Figures S3, S4, S5, S6). The subsetted core-genome and pangenome maximum-likelihood and neighbour-joining trees (Figures 5, 6, Supplementary Figures S1, S2) showed that the isolate clustered most closely with human isolates from France, while remaining genetically distinct from them, and was nested within a larger cluster of French isolates derived primarily from humans, along with some from animals (mainly pets). Virtually all isolates in the subclade (human- and animal-derived) were positive for the *scn* gene, which is considered to be a marker of human adaptation (Howden et al., 2023) (Figures 5, 6).

Average nucleotide identity (ANI) analysis showed that the query genome was highly similar to all tightly clustering reference genomes, with ANI values ranging from 99.956 to 99.984% (Supplementary Table S2). For all pairwise comparisons, the majority of genomic fragments were matched (856–862 of 867 fragments). Pairwise SNP distance analysis of the core SNP alignment revealed moderate genetic divergence among the genomes clustering with the Austrian genome (Supplementary Figure S7). The minimum Hamming distances of the closest neighbours to the studied isolate ranged from 148 to 316 (Supplementary Table S3).

In short, the Austrian MSSA ST398 isolate was genetically related to other MSSA ST398 human and animal isolates from France that also encoded the staphylococcal complement inhibitor SCIN.

## 4 Discussion

MSSA ST398 is an animal-independent lineage of the pandemic clonal complex CC398, which has been reported worldwide and is implicated in severe infections in humans (Bouiller et al., 2020). The phage-encoded immune evasion cluster that contains the *scn* gene, which mediates human adaptation and community spread, is believed to be the major factor behind its rapidly increasing prevalence (Annavajhala et al., 2022; David et al., 2013; Uhlemann A. et al., 2013; Thammavongsa et al., 2015; Uhlemann et al., 2012). In this study, we report for the first time the presence of MSSA ST398 in the community of Vienna, Austria. The isolate, which belongs to a previously undescribed *spa* type, was recovered from a private household and was most closely related to French human and animal isolates, almost all of which encoded the *scn* gene in their prophages. While there appears to be a consensus in the literature on the important role of the community and households in the transmission of MSSA ST398 specifically (especially in contrast to MSSA ST1898) (Annavajhala et al., 2022; Bhat et al., 2009; Uhlemann et al., 2012), recent studies have called for a reassessment of the *scn* gene as a unique marker of human adaptation, as it has been frequently detected in animals, particularly pets (Tegegne et al., 2022). Although a single isolate found in a private Austrian household is insufficient to make broader conclusions about the distribution of MSSA ST398 in Austria, or the role of the community and households as reservoirs, this finding provides a testable hypothesis. It should, therefore, become the goal of follow-up studies to systematically perform typing of MSSA isolates from both community and clinical settings in Austria to obtain information on its true prevalence. This is especially important in light of the fact that MSSA ST398 spreads not only in the community but also in hospital settings, where it was found to be a negative predictor of survival for patients with bloodstream infections (Bouiller et al., 2016), and a spillover from the community appeared to be the culprit behind the MSSA ST398 spread in a neonatal intensive care unit (Annavajhala et al., 2022). Moreover, as shown previously, prevalence can vary a lot even between neighbouring countries such as France (approximately 20%) and Germany (0.13–0.14%) (Bouiller et al., 2020; Cuny et al., 2013); therefore, accurate data on local prevalence are essential. In our phylogenetic analyses, we demonstrated that the Austrian isolate clusters most closely with human and animal (mainly pet-derived) MSSA ST398 isolates from France, with a high congruence between the two methods (pangenome and core-genome NJ and ML), suggesting that transfer to non-human hosts is possible and may represent an underappreciated source of MSSA ST398. This is similar to the findings of the expanding host range of *S. aureus* despite its host specificity, demonstrated in a large phylogenetic study of isolates spanning all One Health sectors (Pan et al., 2023), and multiple reports of shared *S. aureus* strains between owners and pets within the same household (Bethe et al., 2024; Gómez-Sanz et al., 2013a; Wang et al., 2008). However, further research is needed to determine whether animals are persistently or transiently colonised with MSSA ST398 acquired from humans. While it is notoriously difficult to infer the directionality of transmission, our ANI and pairwise SNP distance analyses showed that the Austrian isolate is related to the French isolates with which it clusters. This finding may suggest a possible introduction from neighbouring countries or indicate the circulation of this lineage across the wider European region, with national surveillance systems detecting its presence more effectively in some countries than in others. The core-genome- and pangenome-based maximum-likelihood phylogenies presented here provide a robust framework for assessing genetic relatedness among *Staphylococcus aureus* MSSA strains; however, several limitations must be acknowledged. First, phylogenetic clustering reflects shared evolutionary history, rather than direct transmission events, and therefore, cannot be used to infer transmission routes or directionality between hosts. Second, recombination and horizontal gene transfer, which are frequent in *S. aureus*, may obscure true evolutionary relationships, particularly in pangenome-based analyses that rely on gene presence–absence patterns. Third, differences in genome sequencing depth, assembly quality, annotation accuracy, and gene calling may influence both core-genome alignment and pangenome construction, potentially affecting tree topology. In addition, sampling bias in time, geography, or host origin can limit the representativeness of inferred relationships. Finally, pangenome-based phylogenies emphasise accessory genome variation, which may reflect ecological adaptation, rather than vertical inheritance. Consequently, these phylogenetic reconstructions should be interpreted as measures of genetic similarity, rather than definitive evidence of epidemiological linkage or transmission.

Interestingly, virtually all human- (except one isolate) and animal-derived MSSA ST398 genomes that clustered with the Austrian isolate in our phylogenetic analyses contained the *scn* gene in their prophages. The staphylococcal complement inhibitor protein SCIN, encoded by *scn*, has been shown to associate with C3 convertase from humans but not from other vertebrates (Jongerius et al., 2007), while the phage-encoded immune evasion cluster (IEC) that harbors the *scn* gene has often (but not always) been found in many clinical human isolates (Thammavongsa et al., 2015), including human MRSA ST398 isolates, which have re-acquired the IEC (typically lost in livestock-associated MRSA ST398) (Sieber et al., 2020). Based on this evidence, the *scn* gene has been considered a marker of the phage-encoded IEC1 associated with human adaptation (Aerts et al., 2022). Nonetheless, our findings and those of others (Tegegne et al., 2022) show that the *scn* gene can also be detected in animal isolates of MSSA ST398, highlighting the importance of adopting a One Health approach to its surveillance. Surveillance efforts can be aided by *spa* typing, which distinguishes isolates based on short-sequence repeats (SSRs) in the polymorphic X region of the staphylococcal protein A gene (*spa*) (Frénay et al., 1996). Our *spa* typing analysis showed that the isolate belongs to a previously undescribed type with the succession sequence 08–83–34-25, with the closest spa repeats are 08-83-34-24 from the t16361 type, which has previously been reported in a survey of MRSA from hospitals in Kuwait (Boswihi et al., 2020). *Spa* type t16361 (succession of repeat codes: 08-83-34-24) and the sequence identified in our strain (08-83-34-25) differ by a single repeat unit, and are therefore, potentially related and can share a close evolutionary relationship. The identification of novel *spa* types is not uncommon (Abdulkadir et al., 2024; Reischauer et al., 2010) and may indicate the evolution of the local population in a specific area. Indeed, while our strain clustered most closely with isolates from France, it was genetically distinct from them (Figures 5, 6). Large-scale monitoring of MSSA ST398 in Austria and *spa* typing should help shed light on the existence of a specifically local Austrian *spa* type.

Similar to other MSSA ST398 isolates, our strain contained the *ermT* gene, which mediates resistance to lincosamide (such as clindamycin) and macrolide (such as erythromycin) antibiotics and was also found on a plasmid (Gómez-Sanz et al., 2013b; Salgueiro et al., 2023). Antimicrobial susceptibility testing confirmed that the isolate was indeed resistant to clindamycin and erythromycin, which is concerning. Clindamycin is the first-choice antibiotic for the treatment of bone infections, while the macrolide antibiotic clarithromycin is used as a second choice for the treatment of severe cases of community-acquired pneumonia. Macrolides belong to the “Watch” group of antibiotics according to the WHO AWaRe classification; and therefore, should be restricted for use and prioritised for the monitoring of resistance (WHO, 2023). While there was good congruence between the genotypic and phenotypic data for resistance to macrolide and lincosamide antibiotics, the remaining identified ARGs using the CARD database (Jia et al., 2017; McArthur et al., 2013) (Figure 2) corresponded to wild-type variants in *S. aureus* (Salgueiro et al., 2023; Fu et al., 2016)^10^, which were not implicated in phenotypic resistance, and in line with our phenotypic susceptibility testing results. Moreover, we found discrepancies between the number of ARGs identified by different databases. For example, using the CARD database (Jia et al., 2017; McArthur et al., 2013), we were able to identify 14 ARGs, whereas the ResFinder database (Zankari et al., 2012) showed only the presence of a single ARG (*ermT*). These dissimilarities between the databases likely derive from differences in the interpretation of wild-type core genes as ARGs by the CARD database.

The MSSA ST398 isolate from our study encoded multiple virulence-associated genes. Importantly, many of them were located on a prophage, and therefore, have the potential to be mobilised: *chp* encoding the chemotaxis inhibitory protein CHIPS (De Haas et al., 2004), and the *scn* gene encoding the staphylococcal complement inhibitor SCIN (Rooijakkers et al., 2007), both of which are involved in immune evasion; as well as two beta-hemolysin genes (*hlb_1* and *hlb_2*) involved in the promotion of skin colonisation (Katayama et al., 2013). The prophage was most closely related to the *Staphylococcus* phage StauST398-4 (NCBI Reference Sequence: NC_023499.1), which belongs to the ϕ3 prophage type typical of the human clade of CC398 and was found in isolates from human invasive infections in France. In that study, the authors showed that StauST398-4 is defective and suggested that lysogenisation favours colonisation of human hosts (Van der Mee-Marquet et al., 2013).

Biocides are important tools for controlling the spread of *S. aureus*. For example, chlorhexidine and benzalkonium chloride are commonly used for the decontamination of skin, mucous membranes, and various surfaces. Typically, BAC is used at concentrations ranging from 0.01% as a skin antiseptic (Artasensi et al., 2021) to 0.1% for surface disinfection (Merchel Piovesan Pereira and Tagkopoulos, 2019), while CHX antiseptic in-use concentrations vary from 0.3% in wipes to 4% in body wash (Paulson, 1993; Rafferty et al., 2019). However, there have been reports of reduced tolerance in settings where such biocides are used, with *qac* genes frequently present in these isolates (Bondurant et al., 2020; He et al., 2014; Sharaf et al., 2025). In our isolate, we found 13 ARGs, involved in resistance to disinfectants, including *qacD*. Similar to the ARGs for antibiotics, the use of three different databases produced different results, with no overlap between them (Figure 3), suggesting potential issues with database maintenance and curation. The isolate showed a reduced susceptibility to benzalkonium chloride and chlorhexidine (MIC 2 μg/mL) compared to the biocide reference testing strain (MIC 1 μg/mL), which is still below the typical in-use biocide concentrations and the epidemiological cutoff values (ECOFFs) of 8 μg/mL for CHX and 16 μg/mL for BAC, as determined by Morrissey et al. (2014), and therefore, cannot be interpreted as clinically significant. Although the difference in MIC values does not appear to be very pronounced, even a small increase may be relevant for biocides, as their efficacy can be influenced by incorrect dilution, pH, surface type, soiling, or insufficient contact time (Maillard and Pascoe, 2024), further complicating efforts to reduce the spread of this pandemic lineage.

Our study reports the presence of a pandemic human-associated MSSA ST398 isolate, belonging to a previously undescribed *spa* type, in the community in Austria for the first time. Although this finding is preliminary and based on a single isolate, it suggests the potentially broader spread of this lineage that may go undetected by current surveillance systems, which primarily focus on MRSA. The combination of resistance to clinically important antibiotics with increased tolerance to disinfectants used for decontamination is particularly concerning, while its genetic relatedness to other MSSA ST398 isolates from pets suggests a potential exchange between humans and animals. Future research should focus on determining its true prevalence in both community and hospital settings and on implementing the One Health approach.

## Data Availability

The datasets presented in this study can be found in online repositories. The names of the repository/repositories and accession number(s) can be found in the article/Supplementary material.

## Footnotes

1 https://github.com/FelixKrueger/TrimGalore, accessed on 16/05/2024.

2 https://github.com/tseemann/mlst; accessed on 29/05/2024.

3 https://github.com/HCGB-IGTP/spaTyper, accessed on 29/05/2024.

4 http://spaserver2.ridom.de/, accessed on 29/05/2024.

5 https://github.com/tseemann/abricate, accessed on 04/06/2024.

6 https://github.com/bhattlab/BacMetSearch, accessed on 22/06/2024.

7 https://github.com/AMRverse/AMRrules/blob/main/rules/Staphylococcus_aureus.tsv accessed on 06/03/2026.

8 https://github.com/AMRverse/AMRrules/blob/main/rules/Staphylococcus_aureus.tsv accessed on 06/03/2026.

9 https://github.com/AMRverse/AMRrules/blob/main/rules/Staphylococcus_aureus.tsv, accessed on 06/03/2026.

10 https://github.com/AMRverse/AMRrules/blob/main/rules/Staphylococcus_aureus.tsv, accessed on 06/03/2026.
