# Correction: Resistance to clinically important antibiotics and reduced susceptibility to disinfectants in the pandemic ST398 methicillin-susceptible *Staphylococcus aureus* from Austria

**DOI:** 10.3389/fmicb.2026.1978035

**Published:** 2026-10-21

**Authors:** Frontiers Production Office

**Affiliations:** Frontiers Media SA, Lausanne, Switzerland

**Keywords:** AMR, biocide resistance, community, MSSA, ST398

An incorrect Funding statement was provided.

The correct funding statement reads:

