# Supplementary material for: Resistance to clinically important antibiotics and reduced susceptibility to disinfectants in the pandemic ST398 methicillin-susceptible Staphylococcus aureus from Austria
Source: Front Microbiol. 2026 Apr 21;17:1734430. doi: 10.3389/fmicb.2026.1734430 (PMC13139333; doi:10.3389/fmicb.2026.1734430)
Supplement: Supplementary file 1 [file Supplementary_file_1.docx]

Supplementary Material

## Supplementary Figures


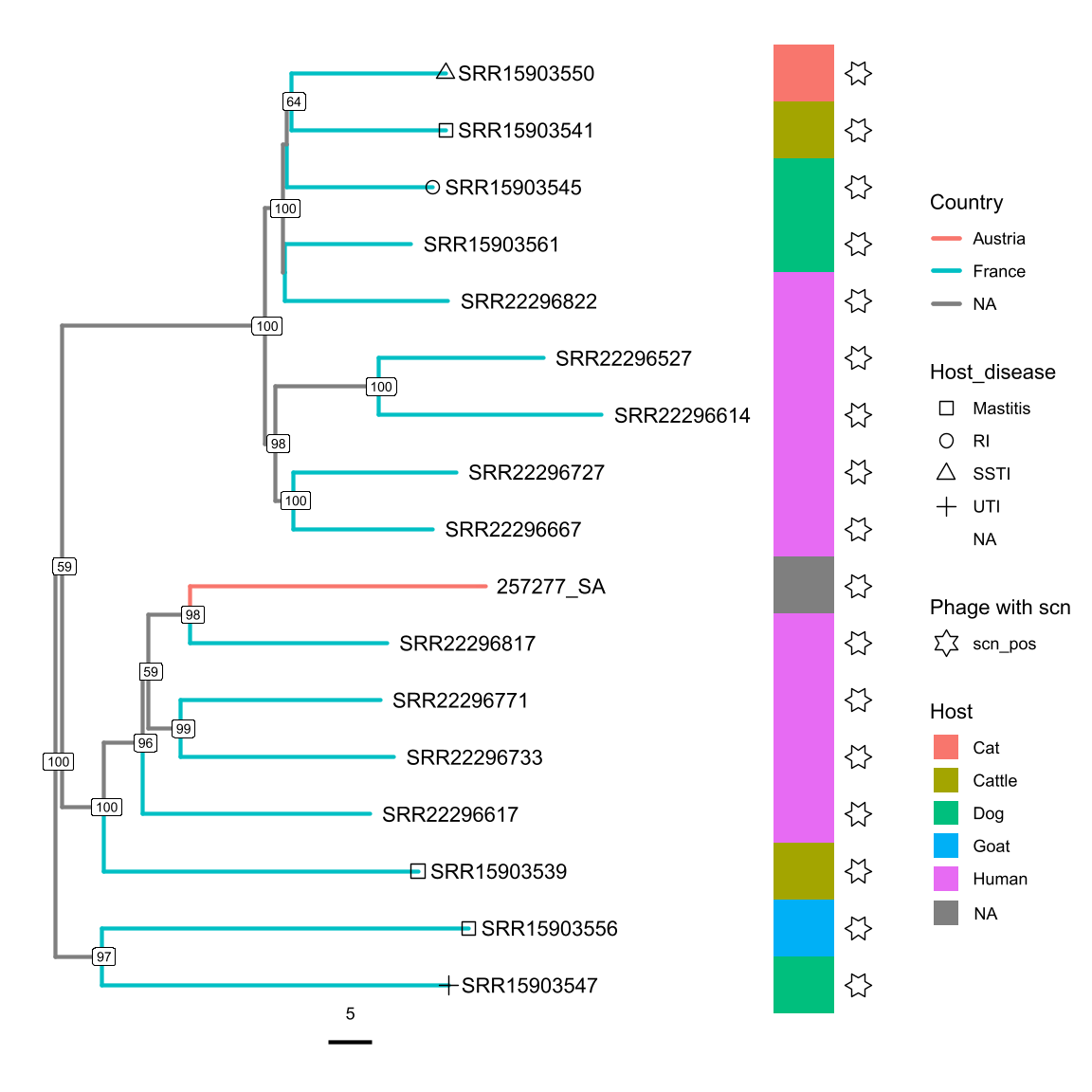


**Supplementary Figure 1**. A zoom in of the core genome neighbor-joining tree. For the full tree see **Supplementary Figure 3**. Host disease abbreviations: RI - Respiratory Infection, SSTI - Skin and Soft Tissue Infection, UTI - Urinary Tract Infection.


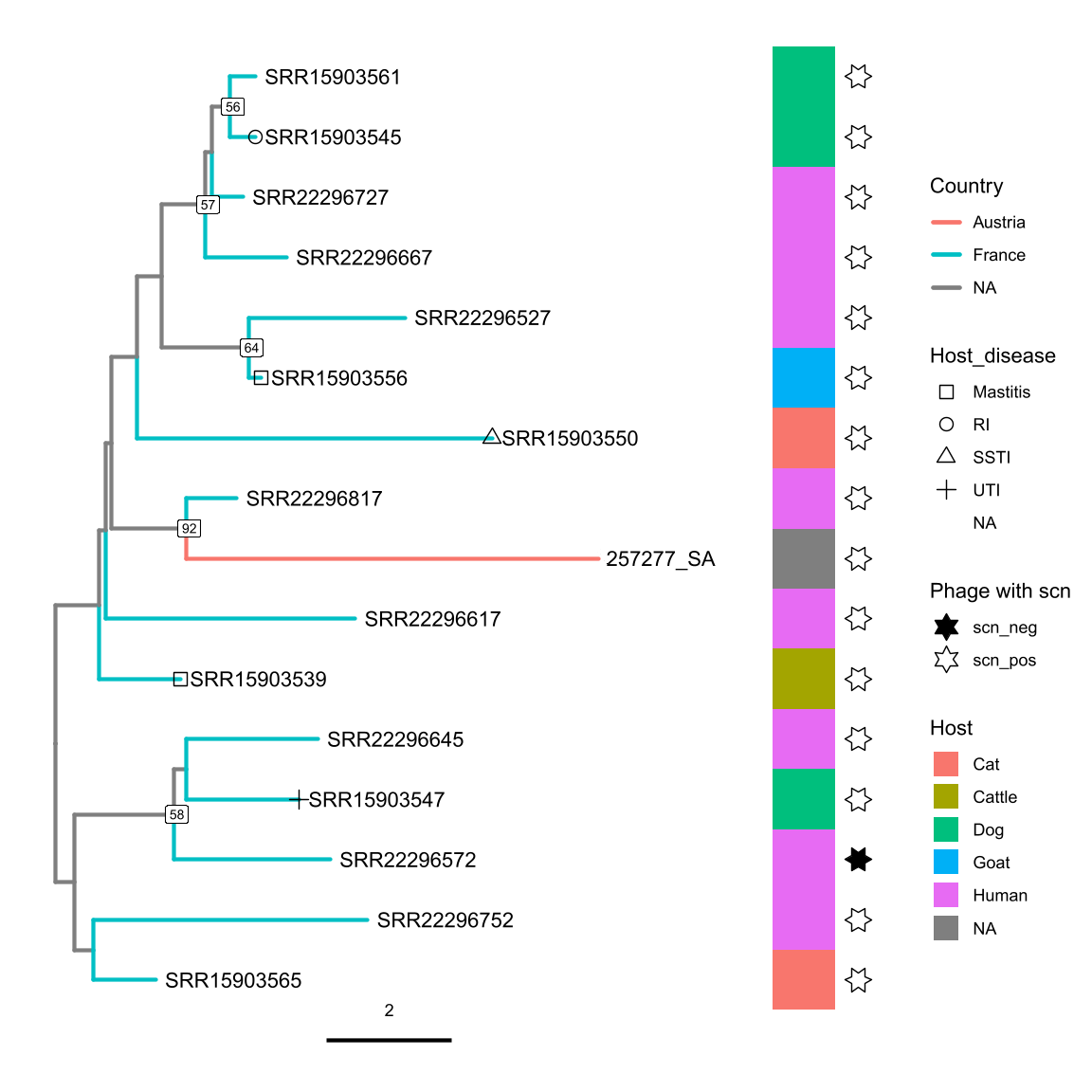


**Supplementary Figure 2**. A zoom in of the pangenome neighbor-joining tree. For the full tree see **Supplementary Figure 4**. Host disease abbreviations: RI - Respiratory Infection, SSTI - Skin and Soft Tissue Infection, UTI - Urinary Tract Infection.

**
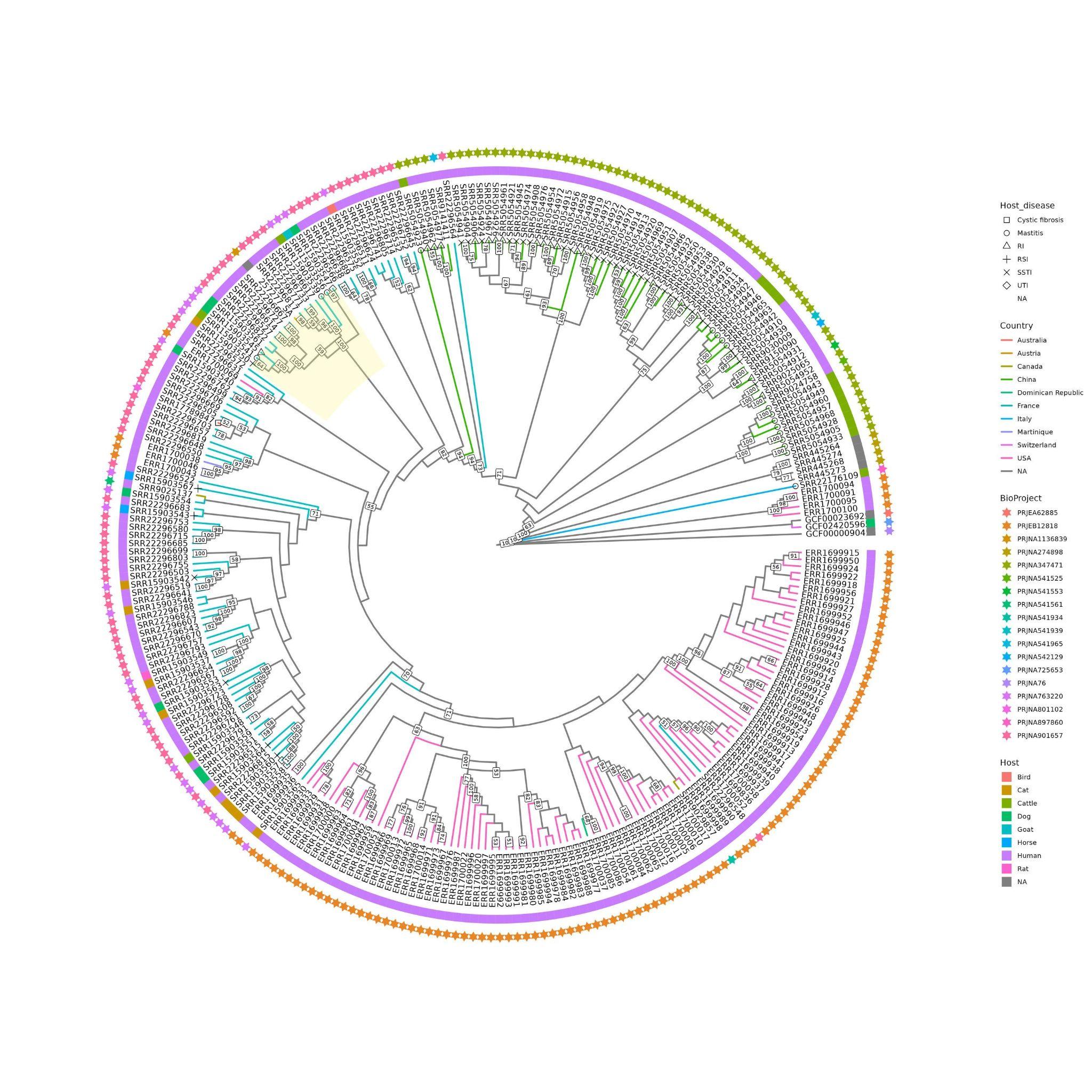
**

**Supplementary Figure 3**. Core genome neighbor-joining cladogram. The area of interest depicted in **Supplementary Figure 1** is highlighted here in yellow. Host disease abbreviations: RI - Respiratory Infection, RSI - Reproductive System infection, SSTI - Skin and Soft Tissue Infection, UTI - Urinary Tract Infection.

**
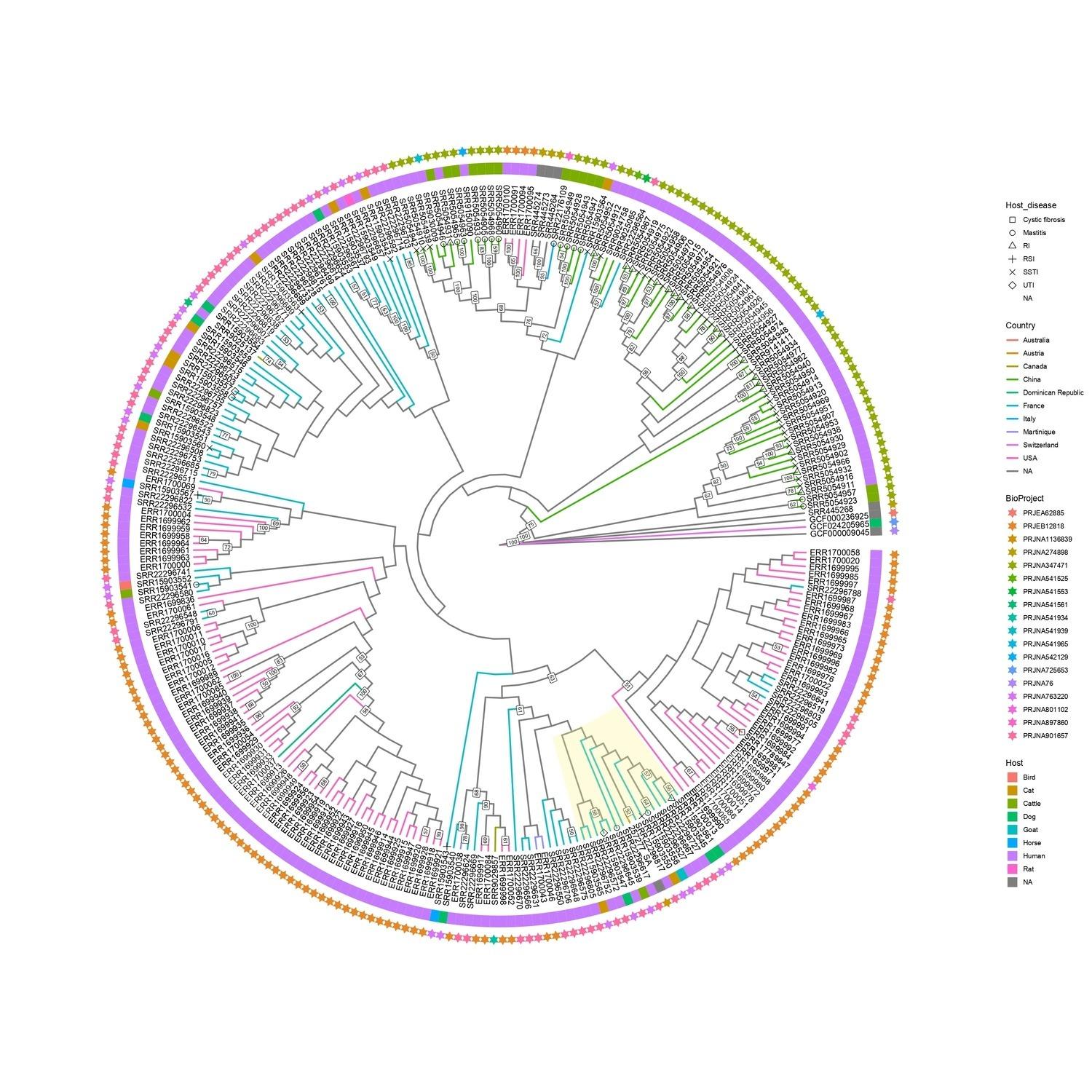
**

**Supplementary Figure 4**. Pangenome neighbor-joining cladogram. The area of interest depicted in **Supplementary Figure 2** is highlighted here in yellow. Host disease abbreviations: RI - Respiratory Infection, RSI - Reproductive System infection, SSTI - Skin and Soft Tissue Infection, UTI - Urinary Tract Infection.


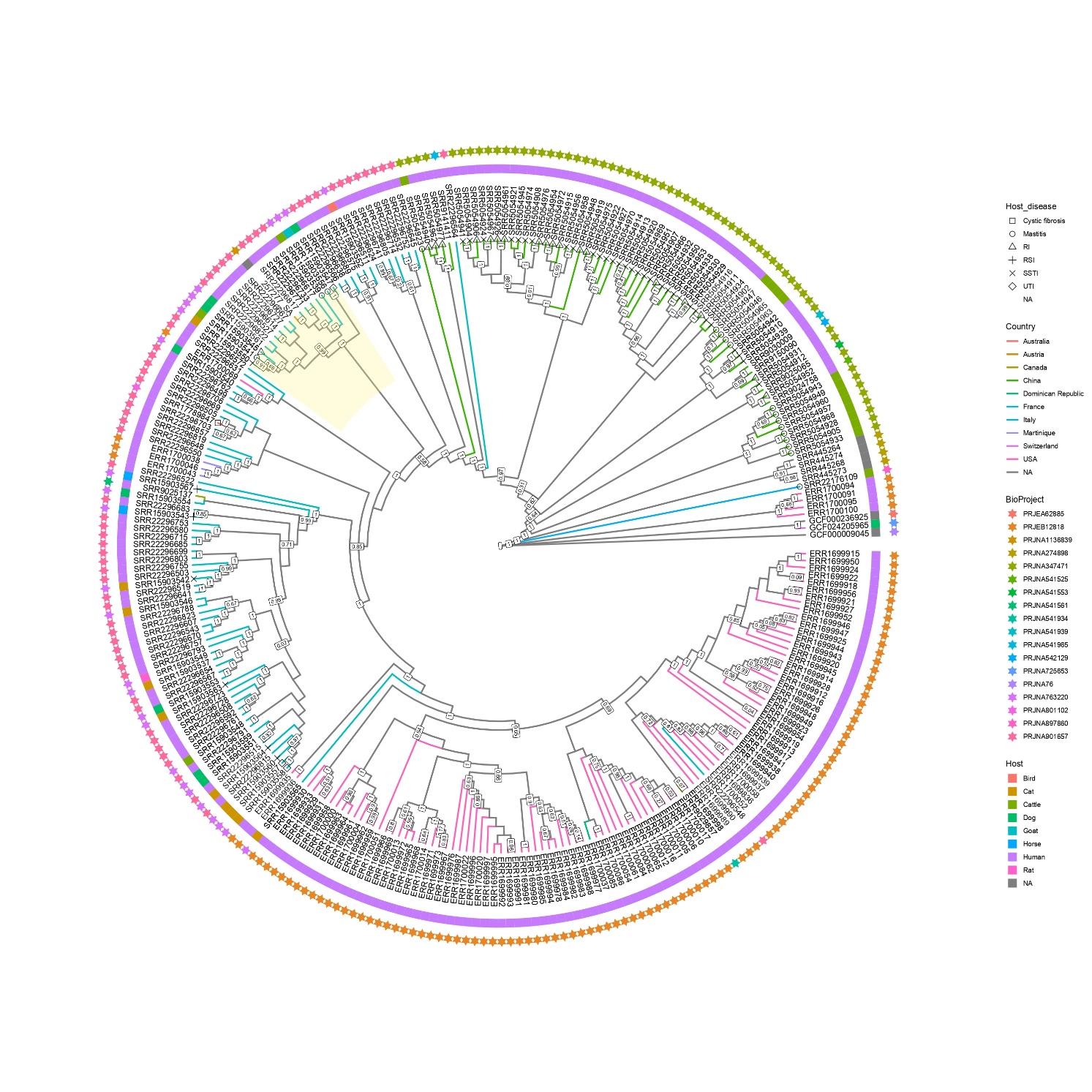


**Supplementary Figure 5**. Core genome maximum likelihood cladogram. The area of interest depicted in **Figure 5** is highlighted here in yellow. Host disease abbreviations: RI - Respiratory Infection, RSI - Reproductive System infection, SSTI - Skin and Soft Tissue Infection, UTI - Urinary Tract Infection.


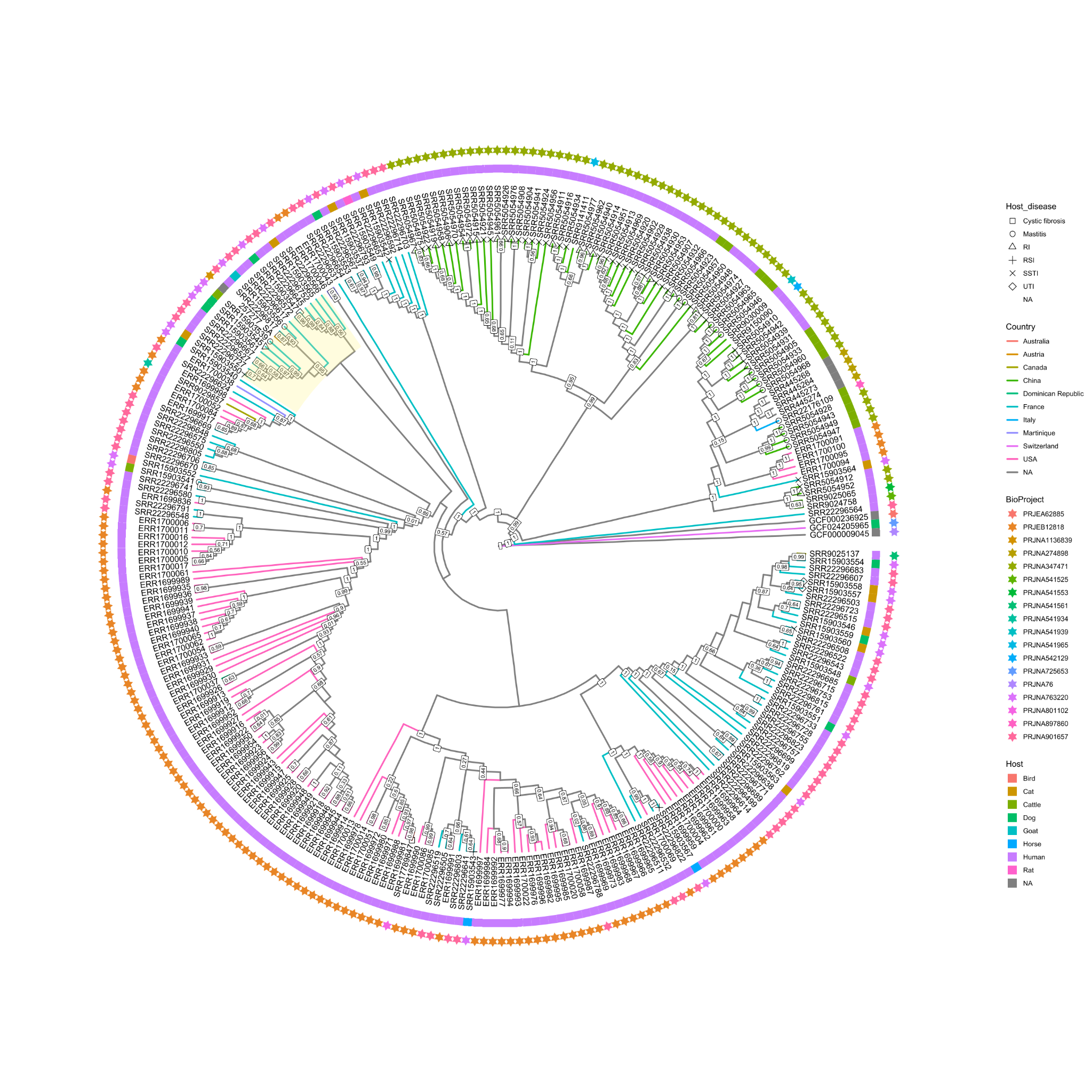


**Supplementary Figure 6**. Pangenome maximum likelihood cladogram. The area of interest depicted in **Figure 6** is highlighted here in yellow. Host disease abbreviations: RI - Respiratory Infection, RSI - Reproductive System infection, SSTI - Skin and Soft Tissue Infection, UTI - Urinary Tract Infection.


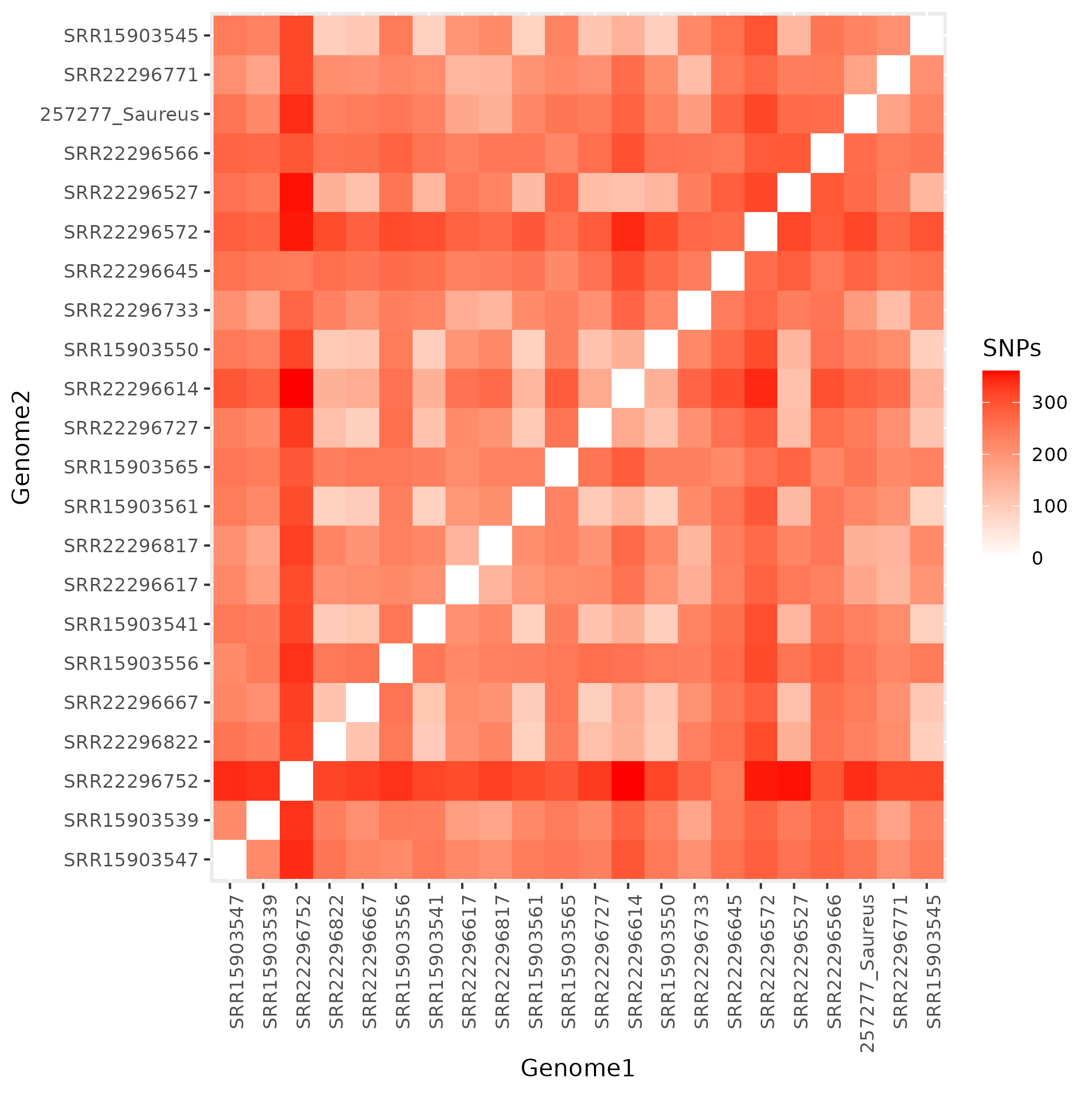


**Supplementary Figure 7**. Pairwise SNP distance analysis of the core SNP alignment of the closest clustering isolates from ML and NJ trees.

**Supplementary Tables**

**Supplementary Table 1.** *Spa* typing results for sequence k141_265, a contig identified in the genome of the *S. aureus* studied. Each row shows the identified *spa* repeat pattern (SPA Pattern Name), its nucleotide sequence (Pattern), strand orientation (Strand), genomic coordinates (Start–End), and the exact matched sequence from the genome (Matched).

| **Contig ID** | **SPA Pattern Name** | **Pattern** | **Strand** | **Start** | **End** | **Matched** |
| --- | --- | --- | --- | --- | --- | --- |
| k141_265 | r08 | GAGGAAGACAACAACAAGCCTGGT | - | 33167 | 33190 | GAGGAAGACAACAACAAGCCTGGT |
| k141_265 | r25 | AAAGAAGATGGCAACAAACCTGGT | - | 33095 | 33118 | AAAGAAGATGGCAACAAACCTGGT |
| k141_265 | r83 | AAAGAAGACGGCAAAAAACCTGGT | - | 33143 | 33166 | AAAGAAGACGGCAAAAAACCTGGT |
| k141_265 | r34 | AAAGAAGACAACAAAAAACCTGGT | - | 33119 | 33142 | AAAGAAGACAACAAAAAACCTGGT |

**Supplementary Table 2**. Average nucleotide identity (ANI) analysis of the core SNP alignment of the closest clustering isolates from ML and NJ trees. The table lists each reference genome (Reference), the calculated ANI percentage (ANI), the number of matched genomic fragments (Matched fragments), and the total number of fragments considered (Total fragments).

| **Query** | **Reference** | **ANI** | **Matched fragments** | **Total fragments** |
| --- | --- | --- | --- | --- |
| 257277_SaureusD1SOM.fa | SRR22296817.fa | 99.9837 | 862 | 867 |
| 257277_SaureusD1SOM.fa | SRR22296617.fa | 99.9743 | 861 | 867 |
| 257277_SaureusD1SOM.fa | SRR15903539.fa | 99.9743 | 861 | 867 |
| 257277_SaureusD1SOM.fa | SRR22296771.fa | 99.9741 | 859 | 867 |
| 257277_SaureusD1SOM.fa | SRR15903545.fa | 99.9735 | 860 | 867 |
| 257277_SaureusD1SOM.fa | SRR15903561.fa | 99.9731 | 859 | 867 |
| 257277_SaureusD1SOM.fa | SRR22296727.fa | 99.9726 | 860 | 867 |
| 257277_SaureusD1SOM.fa | SRR22296733.fa | 99.9706 | 857 | 867 |
| 257277_SaureusD1SOM.fa | SRR22296667.fa | 99.9706 | 861 | 867 |
| 257277_SaureusD1SOM.fa | SRR15903541.fa | 99.9704 | 857 | 867 |
| 257277_SaureusD1SOM.fa | SRR22296527.fa | 99.9698 | 861 | 867 |
| 257277_SaureusD1SOM.fa | SRR15903565.fa | 99.9682 | 858 | 867 |
| 257277_SaureusD1SOM.fa | SRR22296572.fa | 99.9679 | 859 | 867 |
| 257277_SaureusD1SOM.fa | SRR15903547.fa | 99.9669 | 862 | 867 |
| 257277_SaureusD1SOM.fa | SRR15903556.fa | 99.9653 | 862 | 867 |
| 257277_SaureusD1SOM.fa | SRR22296752.fa | 99.9644 | 856 | 867 |
| 257277_SaureusD1SOM.fa | SRR15903550.fa | 99.9632 | 856 | 867 |
| 257277_SaureusD1SOM.fa | SRR22296645.fa | 99.962 | 862 | 867 |
| 257277_SaureusD1SOM.fa | SRR22296614.fa | 99.9585 | 859 | 867 |
| 257277_SaureusD1SOM.fa | SRR22296822.fa | 99.956 | 857 | 867 |

**Supplementary Table 3**. Hamming distances between the studied *Staphylococcus aureus* ST398 genome and its closest neighbouring isolates from the ML core- and pangenome trees. For each genome, the corresponding Hamming distance from the studied strain, the country and the host of origin are shown.

| **Genome** | **Hamming Distance (SNPs)** | **Country** | **Host** |
| --- | --- | --- | --- |
| SRR22296817 | 148 | France | Human |
| SRR22296617 | 165 | France | Human |
| SRR22296771 | 173 | France | Human |
| SRR22296733 | 184 | France | Human |
| SRR15903539 | 215 | France | Cattle |
| SRR15903561 | 220 | France | Dog |
| SRR15903545 | 226 | France | Dog |
| SRR15903550 | 229 | France | Cat |
| SRR22296822 | 230 | France | Human |
| SRR15903541 | 230 | France | Cattle |
| SRR22296667 | 239 | France | Human |
| SRR22296727 | 240 | France | Human |
| SRR15903556 | 247 | France | Goat |
| SRR15903565 | 249 | France | Cat |
| SRR15903547 | 251 | France | Dog |
| SRR22296566 | 262 | France | Human |
| SRR22296527 | 266 | France | Human |
| SRR22296566 | 275 | France | Human |
| SRR22296614 | 277 | France | Human |
| SRR22296572 | 316 | France | Human |
